# Utilizing machine learning to optimize agricultural inputs for improved rice production benefits

**DOI:** 10.1016/j.isci.2024.111407

**Published:** 2024-11-16

**Authors:** Tao Liu, Xiafei Li, Xinrui Li, Zhonglin Wang, Huilai Yin, Yangming Ma, Yongheng Luo, Ruhongji Liu, Zhixin Li, Pengxin Deng, Zhenglan Peng, Zhiyuan Yang, Yongjian Sun, Jun Ma, Zongkui Chen

**Affiliations:** 1 Crop Ecophysiology and Cultivation Key Laboratory of Sichuan Province, Rice Research Institute / State Key Laboratory of Crop Gene Exploration and Utilization in Southwest China, Sichuan Agricultural University, Chengdu 611130, China

**Keywords:** Agricultural science, machine learning

## Abstract

Lower efficiency of agricultural inputs in the four conventional rice planting methods limits productivity and environmental benefits in Southwest China. Thus, we developed a machine-learning-based decision-making system for achieving optimal comprehensive benefits during rice production. Based on conventional benefits for achieving optimal benefits, implemented strategies in these planting methods: reducing N fertilizer by 16% while increasing seed inputs by 9% in mechanical transplanting (MT) method improved yield and environmental benefits; reducing N fertilizer and seed inputs by 10–12% in mechanical direct seeding (MD) method decreased environmental impacts; increasing N-K fertilizers and seed inputs by 15–33% in manual transplanting (MAT) method improved its comprehensive benefits by 7–14%; applying N-P-K fertilizer ratio of 2:1:2 in manual direct seeding (MAD) method enhanced yield. Our study provides strategies for improving benefits in these planting methods, with MT method being more beneficial for optimizing comprehensive benefits, especially in yield and environmental impacts, in Southwest China.

## Highlights

• Comprehensive benefit in mechanical transplanting is the higher
• High N and seed inputs decrease environmental impacts in mechanical direct seeding
• Manual transplanting realized optimal benefits with low production potential
• High N, P, and K inputs in manual direct seeding reduce yield and other benefits

## Graphical abstract

**Figure:**
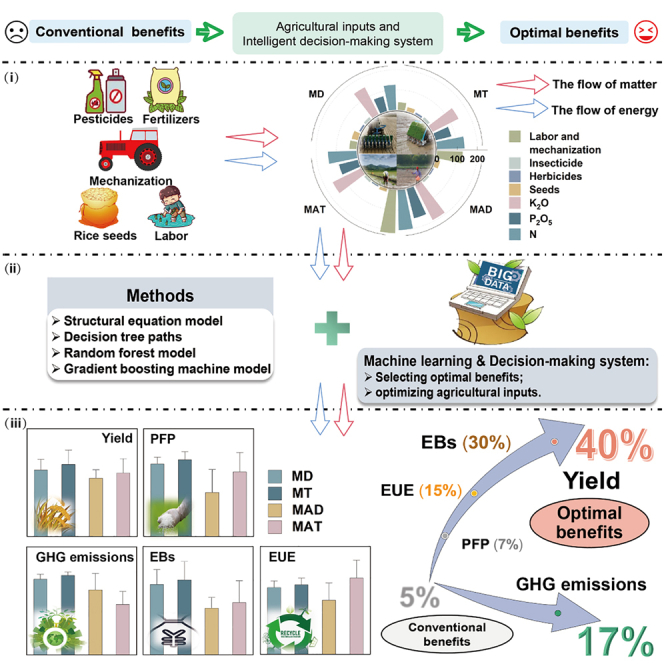

## Introduction

Rice (*Oryza sativa* L.), a vital cereal crop, sustains over half of the global population.^1^ As a major rice-producing country, China relies significantly on the contributions of the Southwest region for its rice cultivation. However, the Southwest region accounts for only 20% of China’s total rice production, which is significantly lower than the rice production in the Northeast and East China regions (https://www.fao.org/faostat/en/). The main planting techniques in Southwest China—mechanical transplanting (MT), mechanical direct seeding (MD), manual transplanting (MAT), and manual direct seeding (MAD)—exhibit approximately 20% inefficiency in converting and utilizing agricultural inputs, presenting substantial hurdles in enhancing production efficiency.^2^^,^^3^ Therefore, exploring the main causes of the low agricultural input transformation efficiency and developing strategies to further improve agricultural inputs and benefits for the four planting methods is crucial for ensuring food security and maintaining sustainable production in Southwest China.

The interaction between the transformation and utilization of agricultural inputs, which include both energy and matter, is crucial in influencing yield, greenhouse gas (GHG) emissions, and economic benefits (EBs) within the rice production system.^4^ In conventional MAT and MAD, the transformation efficiency of agricultural inputs remains at approximately 20–30%. This situation leads to increased soil fertilizer retention, elevated GHG emissions, and higher costs for agricultural inputs, ultimately resulting in lower EBs.^5^ Specifically, increasing conventional fertilizer application by 25%, without adjusting rice seed inputs accordingly, disrupts the nutrient balance, undermining the soil’s nutrient efficacy for rice growth. This imbalance raises input costs and limits potential improvements in grain yield and EBs by approximately 20–40%.^6^^,^^7^ While mechanical planting methods such as MT and MD have become popular, offering a 15% increase in fertilizer utilization efficiency over traditional manual techniques, they still represent emerging agricultural management technologies in the context of rice planting, as the precision of these seeding technologies still needs improvement. This often leads to excessive seed input, making conventional fertilization insufficient to meet the nutritional demands of densely planted rice, thereby reducing growth efficiency and decreasing both grain yield and environmental benefits.^8^ In summary, the fundamental reason for these phenomena is that traditional agricultural management practices are no longer effective in enhancing these benefits. In this context, leveraging machine learning and big data for decision-making emerges as a powerful strategy. This method optimizes agricultural input structures by analyzing key efficiency constraints using advanced models and algorithms. Consequently, it promises to enhance the benefits associated with MT, MD, MAT, and MAD methods, paving the way for the sustainable advancement of rice production.

Machine learning methods excel at identifying the intricate relationships between agricultural inputs and benefits, playing a crucial role in forecasting the developmental trends and pinpointing the current limitations of various rice planting techniques.^9^^,^^10^ The high complexity of support vector machines, artificial neural networks, and genetic programming models reduces their interpretability, making it difficult to determine which agricultural inputs most effectively optimize rice yield, and these models also are highly prone to overfitting, meaning that the predicted optimal agricultural input strategies may be ineffective in real-world applications and cannot be generalized to other fields (https://www.baeldung.com/cs/).^11^^,^^12^^,^^13^ Additionally, recurrent neural networks are only effective for processing continuous information such as time series data.^14^ In contrast, random forest model and gradient boosting machine have been widely applied in agricultural practices due to their high accuracy, interpretability, and low complexity in precision agriculture, and the study indicated that decreasing approximately 12% of GHG emissions without affecting grain yield could be achieved by increasing planting density and reducing N fertilizer application in rice planting process.^15^^,^^16^^,^^17^^,^^18^ These two models can effectively predict and improve agricultural input structures to optimize rice production benefits by generating multiple decision trees based on historical agricultural data. Despite these advances, there remains limited research on how machine learning decision-making systems can optimize agricultural inputs to improve the transformation efficiency of inputs and potential in both manual and mechanical planting methods, which is essential for advancing current agricultural practices toward greater precision and efficiency. Thus, we employed a data-intensive strategy to forge a decision-making system grounded in machine learning, aiming for optimizing agricultural inputs to improve benefits in four rice planting methods, thereby delineating a pathway toward sustainable, low-carbon, and efficient rice production in Southwest China.

## Results

### Differences in the agricultural inputs and benefits of the MT, MD, MAT, and MAD methods

**Figure 1:**
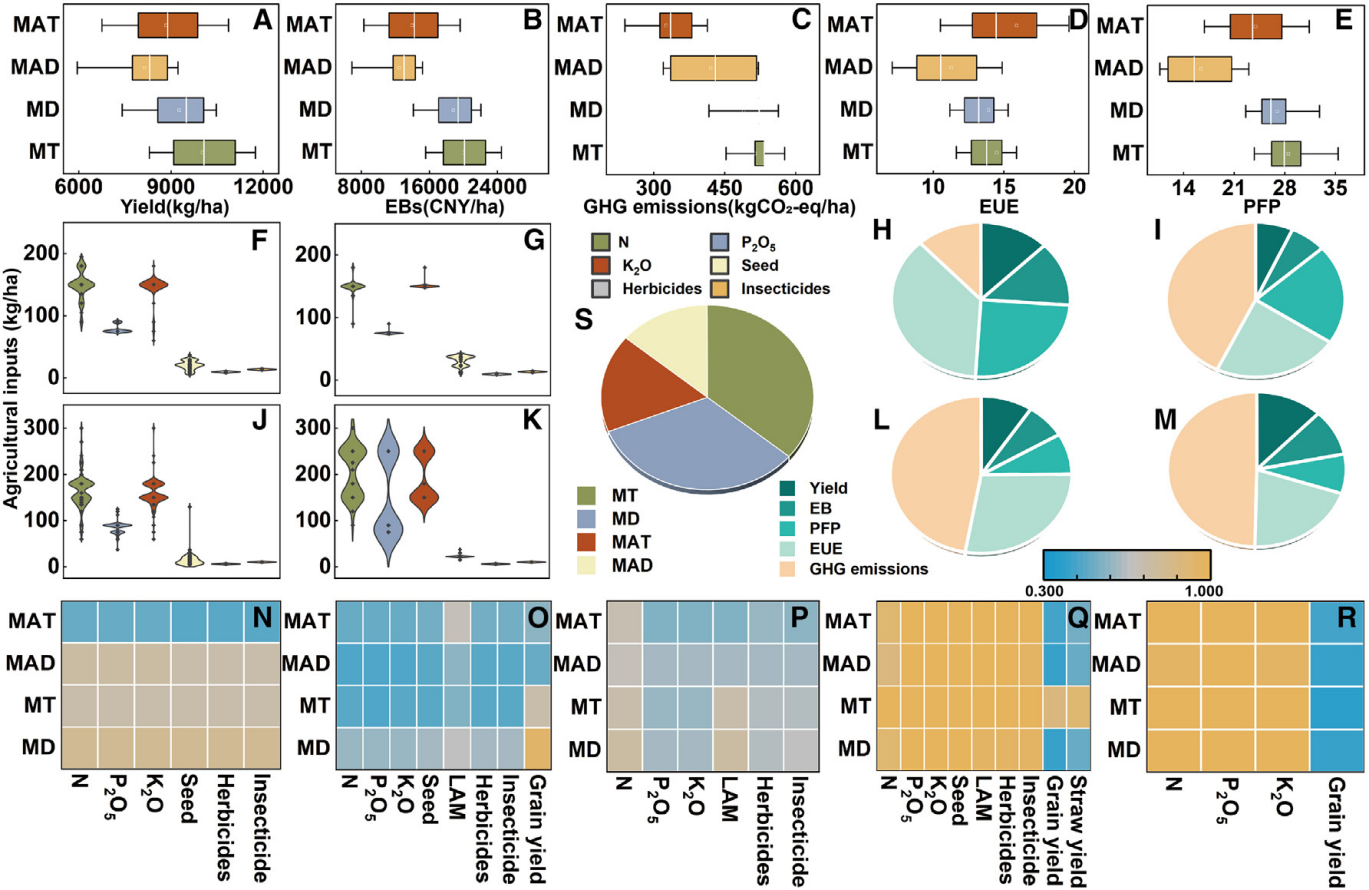
Agricultural inputs and benefits in the four planting methods Yield (A), economic benefits (EBs) (B), greenhouse gas (GHG) emissions (C), partial fertilizer productivity (PFP) (D) and energy use efficiency (EUE) (E) and agricultural inputs (F, G, J, and K) in mechanical transplanting (MT) (*n* = 302), mechanical direct seeding (MD) (*n* = 202), manual transplanting (MAT) (*n* = 1079), and manual direct seeding (MAD) (*n* = 139) methods. Correlation analysis of agricultural inputs and five benefits (N–R). The weight of benefits and composite score (H, I, L, M, and S). Square within the box indicate the mean values, the white line in the box represents the median, box boundaries indicate upper and lower quartiles; bars indicate SD. CNY, Chinese Yuan.

In the MT method, the application rates for N, P, and K fertilizers and seed amounts were 136 kg/ha, 78.6 kg/ha, 143.5 kg/ha, and 20.5 kg/ha, respectively. Relative to the MT method, the MD method used 8% less N and 3.7% less P, but 5% more K fertilizer and 49.9% more seed amounts. In contrast, the MAT method saw increases of 3.7% in N, 21.8% in P, and 9.1% in K, while seed use decreased by 26.3%. In the MAD method, the use of N, P, and K fertilizers increased by 47%, 92.8%, and 34%, respectively (Figure 1). The MT method’s yield, GHG emissions, EBs, partial fertilizer productivity (PFP), and energy use efficiency (EUE) were 1.1 × 10^4^ kg/ha, 514.61 kg CO_2_-eq/ha, 2.1 × 10^4^ yuan/ha, 28.51, and 14.49. These values were 1.08, 1.05, 1.06, 1.06, and 1.04 times greater than those of the MD method; 1.14, 1.58, 1.43, 1.19, and 0.91 times greater than those of the MAT method; and 1.23, 1.22, 1.61, 1.74, and 1.29 times greater than those of the MAD method, respectively.

### Comprehensive benefit analysis of the MT, MD, MAT, and MAD methods

The TOPSIS method indicated that the comprehensive benefits of the MT, MD, MAT, and MAD methods were 38.51, 35.03, 18.34, and 14.81, respectively, with the MT method exhibiting the highest comprehensive benefit (Figure 1). An analysis based on the entropy weighting method revealed that GHG emissions, EUE, yield, PFP, and EBs significantly influenced the comprehensive benefits of the MT and MD methods, in descending order. For the MAT method, the order of contribution was EUE, PFP, EBs, yield, and GHG emissions, whereas for the MAD method, it was GHG emissions, EUE, PFP, yield, and EBs. Among these factors, N fertilizer application was the largest contributor to GHG emissions. The economic income derived from grain yield was the primary supporter of EBs, and the energy output from grain yield formation was most closely correlated with EUE.

### Classification of benefit intervals and their agricultural inputs in the MT, MD, MAT, and MAD methods

**Figure 2:**
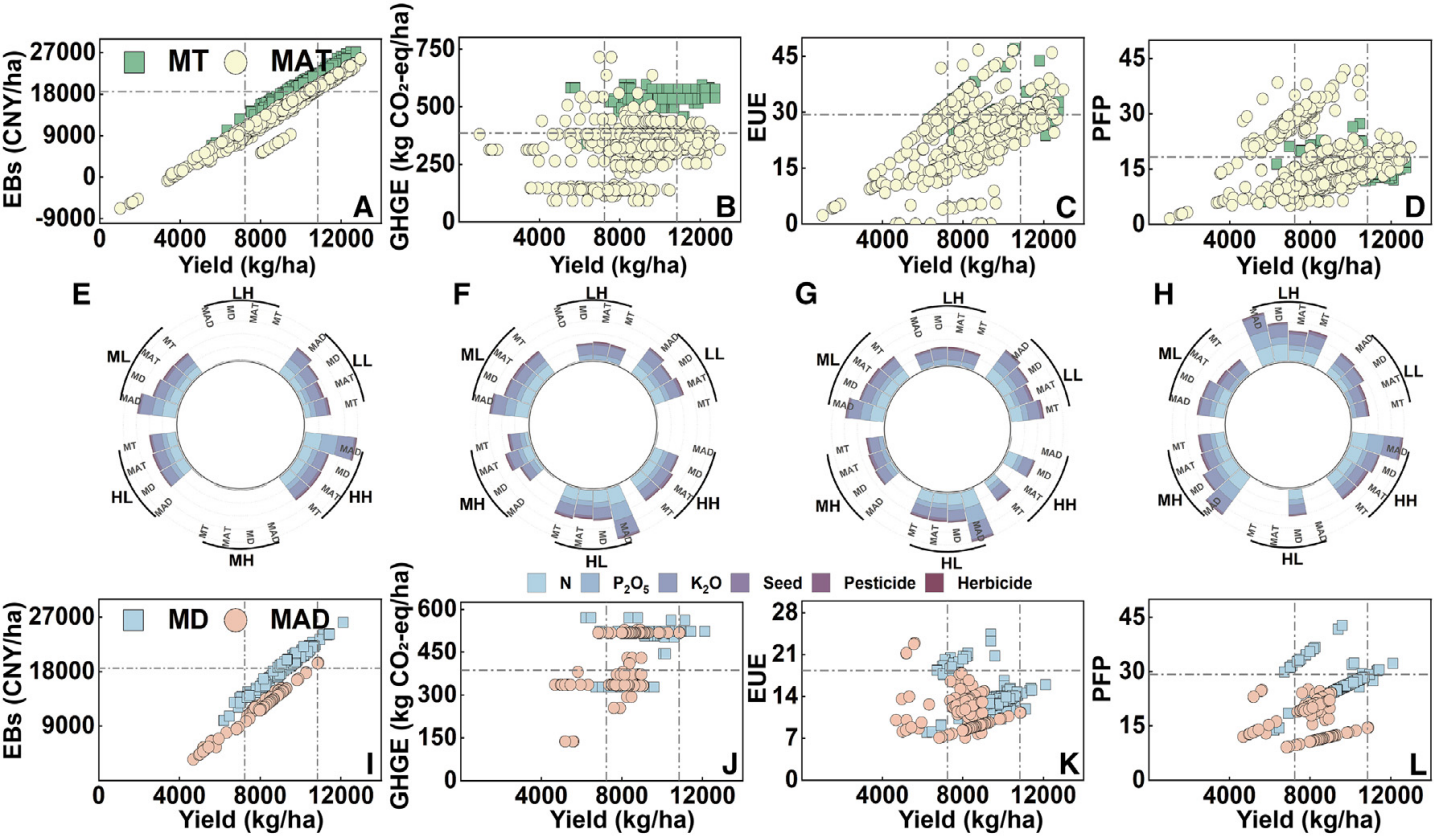
Different benefit intervals and their agricultural inputs in the four planting methods The economic benefits (EBs) (A and I), energy use efficiency (EUE) (B and J), greenhouse gas emissions (GHGE) (C and K) and partial fertilizer productivity (PFP) (D and L) in high yield, middle yield and low yield and agricultural inputs (E–H) of mechanical transplanting (MT) (*n* = 302), mechanical direct seeding (MD) (*n* = 202), manual transplanting (MAT) (*n* = 1,079) and manual direct seeding (MAD) (*n* = 139) methods. The HH, MH, or LH refer to realized high EB, GHG emissions, PFP and EUE in high, middle or low yield of different planting methods, respectively; HL, ML or LL refer to achieved low EB, GHG emissions, PFP and EUE in high, middle or low yield of different planting methods, respectively. CNY: Chinese Yuan.

In the MT, MD, MAT, and MAD methods, the benefit intervals were classified according to high, medium, and low yield; high and low GHG emissions; high and low EBs; high and low EUE values; and high and low PFP values, and their agricultural inputs are shown in Figure 2.

### Differences in agricultural inputs and benefits between conventional, suboptimal, or optimal benefits among the MT, MD, MAT, and MAD methods

**Figure 3:**
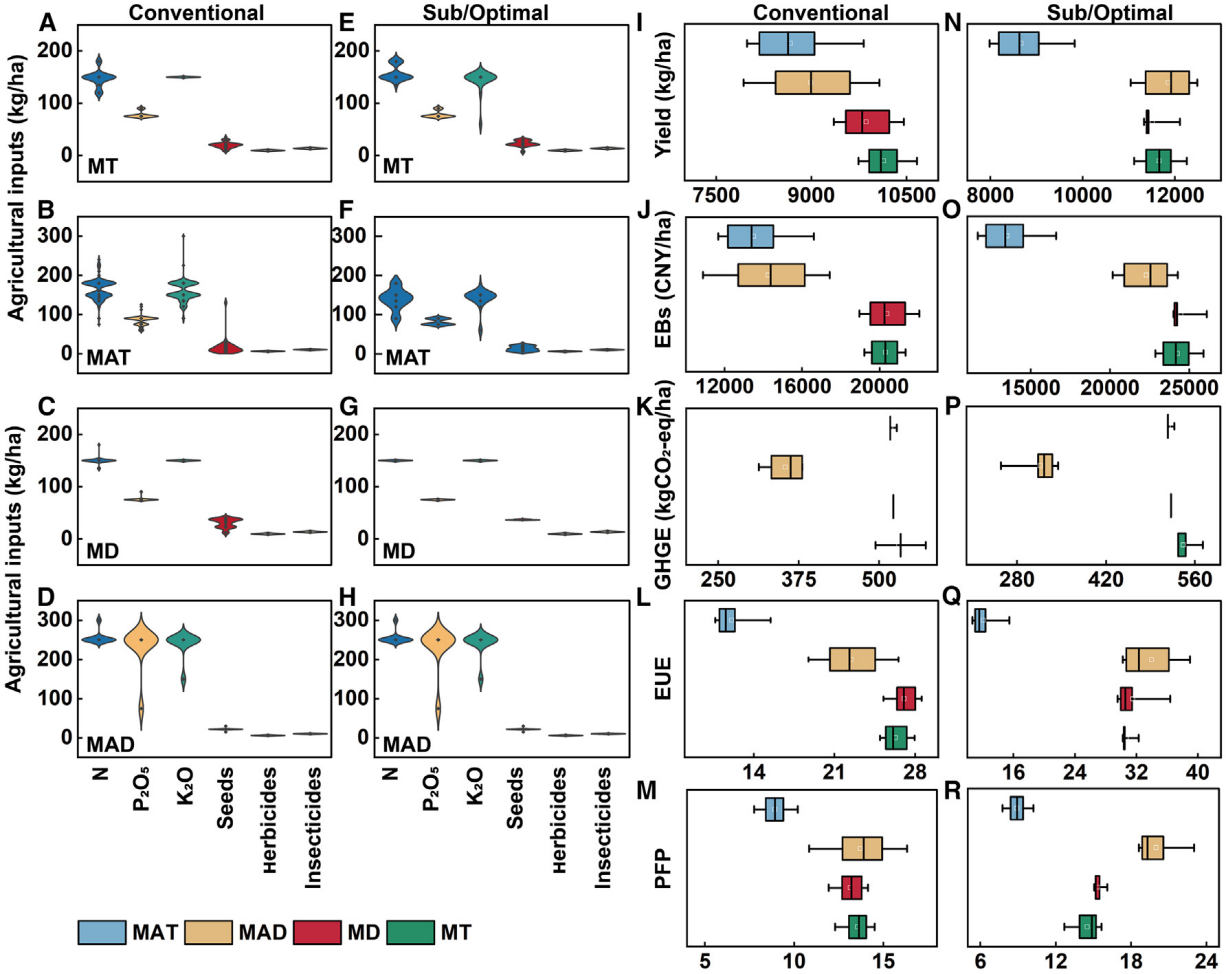
The conventional and suboptimal or optimal benefits in the four planting methods Note: The agricultural inputs and conventional benefits (A–D and I–M) of mechanical transplanting (MT) (*n* = 302), mechanical direct seeding (MD) (*n* = 202), manual transplanting (MAT) (*n* = 1079), and manual direct seeding (MAD) (*n* = 139) methods, the suboptimal or optimal benefits (E–H and N–R) of MT, MD, MAT, and MAD. Square within the box indicate the mean values, the black line in the box represents the median, box boundaries indicate upper and lower quartiles; bars indicate standard deviation.

The conventional benefits of the MT and MD methods included medium yield, high GHG emissions, high EBs, low PFP, and low EUE (MY-HG-HB-LP-LE). The conventional benefits of MAT and MAD methods included medium yield, low GHG emissions, low EBs, low PFP, and low EUE (MY-LG-LB-LP-LE). Compared to the conventional benefits of each planting method, the MT method increased by 7.1% N fertilizer and decreased by 9.1% application of K fertilizer; the MD method by increasing N fertilizer and the seed amounts by 3% and 22%, both realized a shift to suboptimal benefits: high yield, high GHG emissions, high EBs, high PFP, and low EUE (HY-HG-HB-HP-LE) (Figure 3). By reducing 14.7%, 16.5%, and 15.6% of N and P fertilizers and seed amount in the MAT method could realize the optimal benefits of high yield, low GHG emissions, high EBs, high PFP, and high EUE (HY-LG-HB-HP-HE); however, the MAD method did not result in changes from conventional to suboptimal or optimal benefits.

In addition, the suboptimal or optimal benefit benefits can be achieved in the MT, MD, and MAT methods, in the HY-HG-HB-HP-LE of the MT method, yield, EBs, and PFP increased by 15%, 19.4%, 16.6%, but GHG emissions and EUE decreased by 2.1% and 7.2%. In the HY-HG-HB-HP-LE of MD method, the yield, EBs, PFP increased by 16.7%, 16.8% and 16.8%, while GHG emissions also increased by 20%. For the MAT method, the optimal benefits of HY-LG-HB-HP-HE increasing by 31.9%, 56.5%, 51.4%, 45.8% of yield, EBs, PFP, EUE and reducing 10.9% of GHG emissions.

### Relevance of agricultural inputs between the conventional and suboptimal or optimal benefits of the MT, MD, MAT, and MAD methods

**Figure 4:**
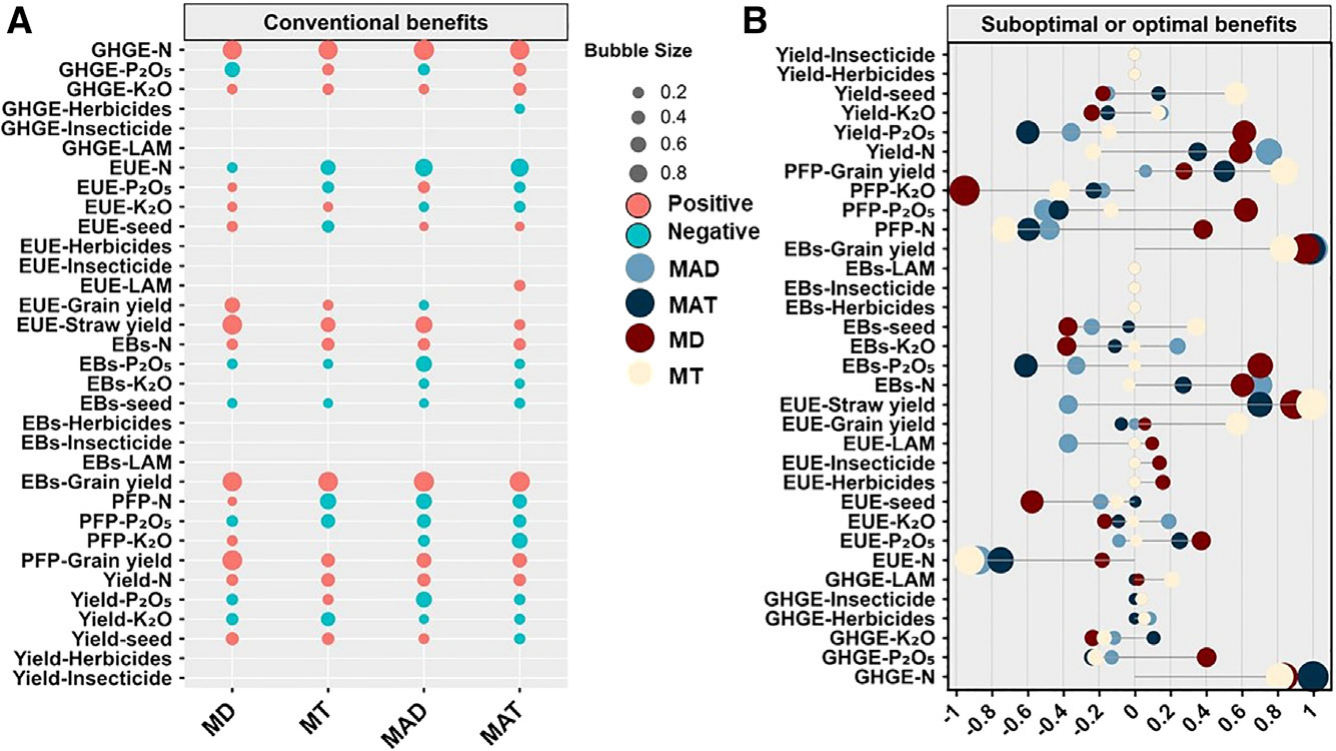
The path analysis between agricultural inputs and benefits using a structural equation model in the four planting methods Correlation analysis of conventional (A) and suboptimal or optimal benefits (B) of agricultural inputs with yield, greenhouse gas emissions (GHGE), partial fertilizer productivity (PFP), energy use efficiency (EUE) and economic benefits (EBs) in mechanical transplanting (MT) (*n* = 302), mechanical direct seeding (MD) (*n* = 202), manual transplanting (MAT) (*n* = 1,079), and manual direct seeding (MAD) (*n* = 139) methods.

Compared with the conventional benefits of each planting method, the HY-HG-HB-HP-LE of the MT and MD method was determined by higher yield, EBs and EUE, and these benefits were supported by N and K fertilizer and seed amounts (Figure 4). The relevance between N fertilizer, yield, and EBs was increased, and although the relevance between N fertilizer and GHG decreased, it did not significantly change the GHG emissions. The HY-LG-HB-HP-HE of the MAT method, N, P, and K fertilizer supported the formation of higher yield and EBs with increased correlation. And the relevance of grain yield with EUE and PFP also increased; however, the MAD method did not result in changes from conventional to suboptimal or optimal benefits.

### Application of machine learning

#### MT, MD, MAT, and MAD methods for decision tree path analysis

**Figure 5:**
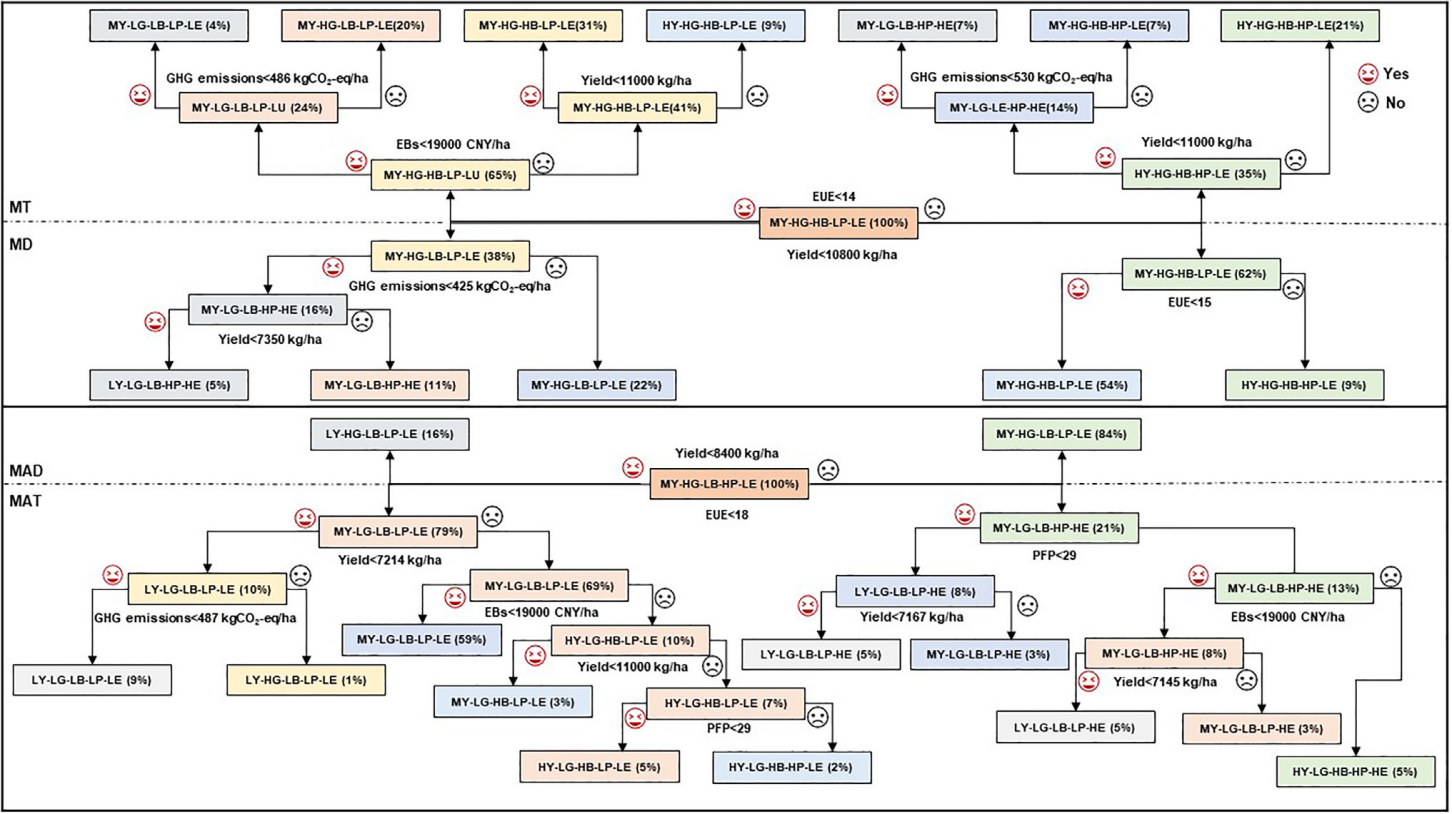
The approaches of achieving suboptimal or optimal benefits basing on decision tree paths in the four planting methods The decision tree for realizing the optimal benefit characteristics in mechanical transplanting (MT) (*n* = 302), mechanical direct seeding (MD) (*n* = 202), manual transplanting (MAT) (*n* = 1,079) and manual direct seeding (MAD) (*n* = 139) methods. The abbreviation combinations refer to high (HY), middle (MY), or low (LY) yield - high (HG) or low (LG) GHG emissions - high (HB) or low (LB) EBs - high (HP) or low (LP) PFP - high (HE) or low (LE) EUE, respectively. The percentage in brackets is the possibility of realizing the benefit characteristics basing on the judgment conditions.

Based on the conventional benefits of MY-HG-HB-LP-LE and by adjusting the yield to ≥1.1 × 10^4^ kg/ha, GHG emissions to ≥530.8 kgCO_2_-eq/ha and EUE ≥14, the MT method could result in the suboptimal benefits of HY-HG-HB-HP-LE (21%) (Figure 5). Based on the conventional benefits of MY-HG-HB-LP-LE and by adjusting the yield to ≥1.08 × 10^4^ kg/ha and the EUE to ≥15, the suboptimal benefits of the MD method were HY-HG-HB-HP-LE (9%). Based on the conventional benefits of MY-LG-LB-LP-LE and by setting the EUE to ≥18, the PFP to ≥29, and the EBs to ≥1.9 × 10^4^ CNY/ha, the MAT method would result in the optimal benefits of HY-LG-HB-HP-HE (5%). The MAD method, did not achieve optimal benefits due to a yield limitation of ≤8.4 × 10³ kg/ha.

#### Analysis of better benefits in the MT, MD, MAT, and MAD methods using a random forest model and gradient boosting machine

**Figure 6:**
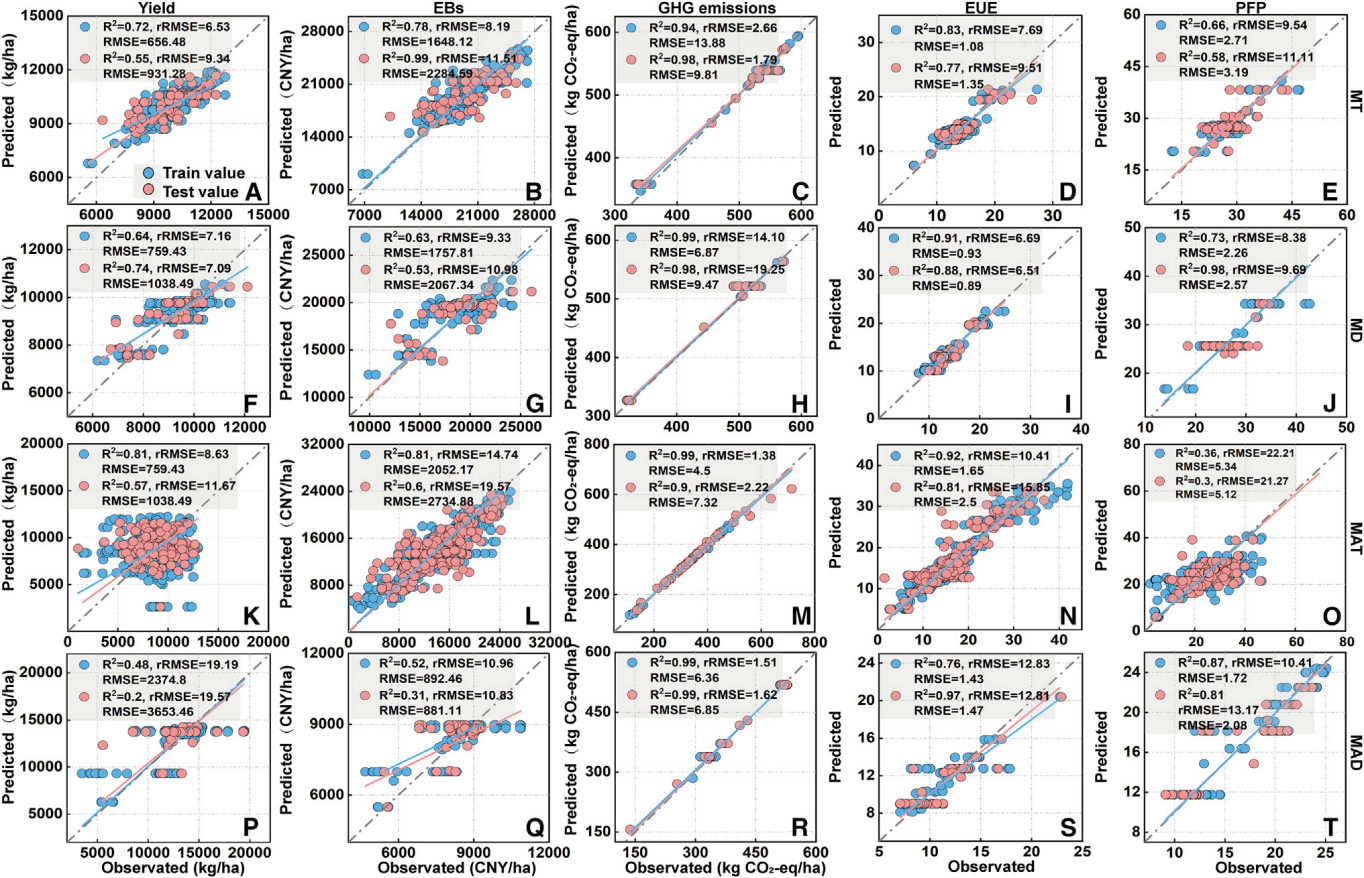
The test of random forest model in the four planting methods The presentation of measured and observed values for yield, economic benefits (EBs), greenhouse gas (GHG) emissions, partial fertilizer productivity (PFP), and energy use efficiency (EUE) (A–T) in mechanical transplanting (MT) (*n* = 302), mechanical direct seeding (MD) (*n* = 202), manual transplanting (MAT) (*n* = 1079), and manual direct seeding (MAD) (*n* = 139) methods. Where blue color represents the results predicted from the training data, and red color represents the results predicted from the testing data.

**Figure 7:**
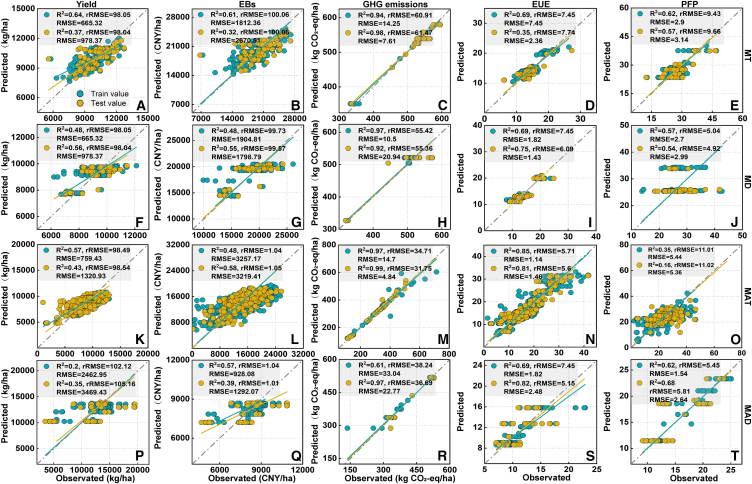
The test of gradient boosting machine in the four planting methods The presentation of measured and observed values for yield, economic benefits (EBs), greenhouse gas (GHG) emissions, partial fertilizer productivity (PFP), and energy use efficiency (EUE) (A–T) in mechanical transplanting (MT) (*n* = 302), mechanical direct seeding (MD) (*n* = 202), manual transplanting (MAT) (*n* = 1079), and manual direct seeding (MAD) (*n* = 139) methods. Where purple color represents the results predicted from the training data, and yellow color represents the results predicted from the testing data.

**Figure 8:**
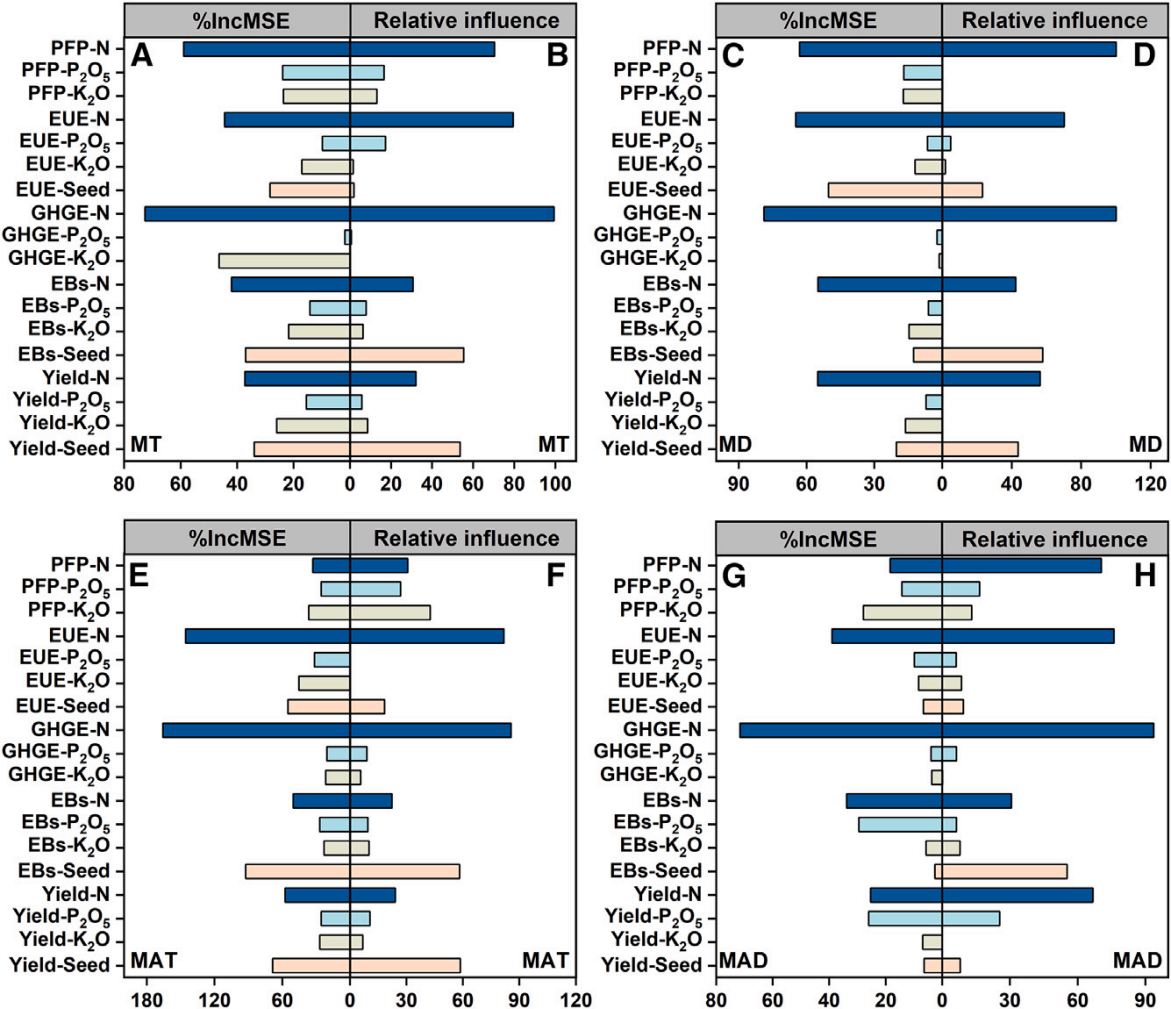
Analysis of the importance of agricultural inputs for benefits in the four planting methods (A, C, E, and G) represent the variable importance of benefits (yield, economic benefits (EBs), greenhouse gas emissions (GHGE), partial fertilizer productivity (PFP), and energy use efficiency (EUE)) as determined by the random forest model, while (B, D, F, and H) represent the relative influence of agricultural inputs on different benefit levels, as assessed by the gradient boosting machine in the mechanical transplanting (MT) (*n* = 302), mechanical direct seeding (MD) (*n* = 202), manual transplanting (MAT) (*n* = 1079) and manual direct seeding (MAD) (*n* = 139) methods.

**Figure 9:**
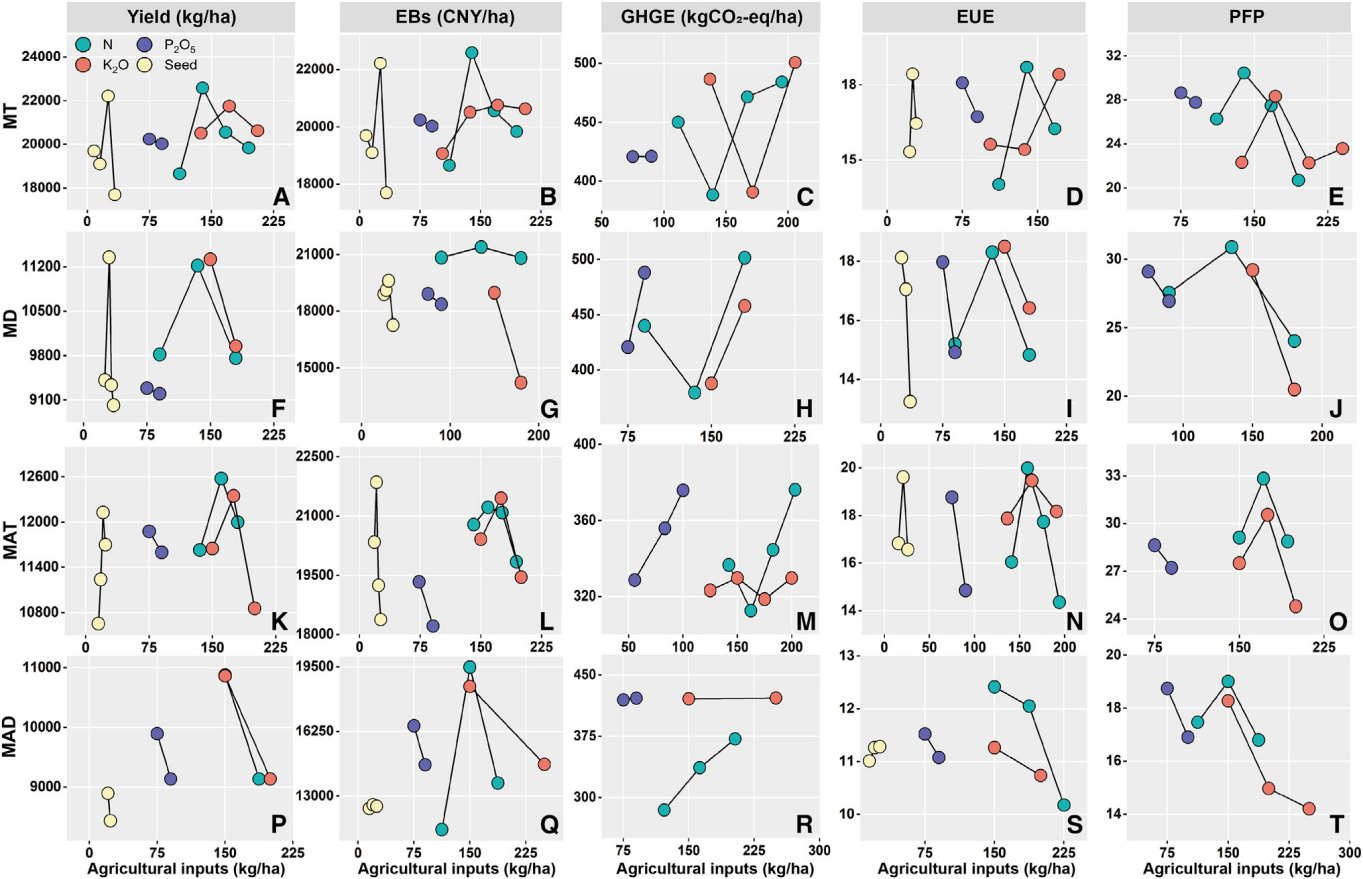
The biased dependence of benefits on agricultural inputs in the four planting methods The graph of the biased dependence (A–T) between agricultural inputs and yield, economic benefits (EBs), greenhouse gas emissions (GHGE), partial fertilizer productivity (PFP) and energy use efficiency (EUE)) in mechanical transplanting (MT) (*n* = 302), mechanical direct seeding (MD) (*n* = 202), manual transplanting (MAT) (*n* = 1079), and manual direct seeding (MAD) (*n* = 139) methods.

The prediction results of two models indicate the importance gradient of agricultural inputs for benefits under different planting methods (Figures 6 and 7): the benefits of MT and MAT methods are strongly associated with N and K fertilizers and seed amounts; the benefits of the MD method are strongly related to N fertilizer, seed amounts, and K fertilizer; and the MAD method benefits are strongly associated with N, P, and K fertilizers (Figure 8). Based on the currently reaching benefits, the optimization results for agricultural inputs across distinct planting methods (Figure 9), given the suboptimal benefits of HY-HG-HB-HP-LE, GHG emissions could be reduced by about 26% and the yield and EUE in the MT method could be increased by about 15 and 30% with no significant impact on other benefits by reducing N fertilizer application by 16% while boosting K fertilizer and seed inputs by 19% and 9%. In the MD method, GHG emissions could be reduced by about 21% and the EUE could be increased by about 32% with no significant impact on other benefits by reducing N fertilizer and seed inputs by 10% and 12%. Together, these changes achieved optimal benefits (HY-LG-HB-HP-HE) for both methods. Based on the optimal benefits, the MAT method could increase yield, EBs, PFP, and EUE by 7%, 14%, 12%, and 9% by increasing N and K fertilizers, and seed amounts by 15%, 30% and 33%, respectively. Finally, based on the conventional benefits of high yield, high GHG emissions, high EBs, low PFP, and low EUE (HY-HG-HB-LP-LE), the MAD method could increase yield and EBs by 33–40% by reducing the N, P, and K fertilizers by 24.24%, 28.51%, and 20.28%.

## Discussion

### Effect of agricultural inputs on the differences in the comprehensive benefits of the MT, MD, MAT, and MAD methods

Variations in agricultural inputs, such as fertilizers and seeds, led to significant differences in the comprehensive benefits observed across the four rice planting methods. Assessing the contributions of these inputs was critical for enhancing yield and optimizing both economic and environmental benefits in rice planting process. In this study, we employed TOPSIS and entropy weighting methods to thoroughly evaluate the MT, MD, MAT, and MAD methods, focusing on key benefit indicators such as yield, GHG emissions, EBs, PFP, and EUE. Among the methods, the MT method demonstrated the highest comprehensive benefits, outperforming the MD, MAT, and MAD methods by 1.1, 2.1, and 2.6 times, respectively. The high yield and EBs achieved by the MT method were attributed to its precise application of N fertilizer and seeds, despite the associated increase in GHG emissions. In contrast, the MD method showed lower comprehensive benefits due to reduced yield, EBs, and GHG emissions, which were linked to its lower rates of N fertilizer and seed inputs. The MAT method resulted in lower yield, EUE, PFP, and EBs due to inadequate regulation of N, P, and K fertilizers, while the MAD method, characterized by excessive fertilizer use, yielded the least balanced benefits, resulting in the lowest comprehensive benefits.

In the MT method, seed and total fertilizer inputs were maintained at 20.5 kg/ha and 353 kg/ha, respectively, significantly enhancing yield and EBs. This strategy enhanced planting density and soil nutrient enrichment, thereby enhancing the production potential of the method. Moreover, increased fertilizer application enhanced root vitality, enabling roots to extract nutrients from deeper soil layers, thus improving nutrient absorption and utilization in rice plant. This process promoted rice tillering (especially, the number of its effective tillers) and the functional leaf growth, thereby boosting photosynthesis in the functional leaves of rice and efficient nutrient accumulation in rice grains—factors that collectively drove up yield and PFP.^19^^,^^20^^,^^21^ Concurrently, these yield improvements also increased the economic returns of growers by counterbalancing the input costs for N, P, and K fertilizers and seeds, thereby achieving the highest EBs. Nonetheless, increased N fertilizer application acidified the soil, suppressing certain N-metabolizing soil microorganisms, increasing N retention in the soil, decreasing the soil C-N ratio, thereby promoting GHG emissions in rice fields.^22^ Additionally, high levels of agricultural mechanization require increased energy input, yet inefficient energy flows during production can result in a 20.8% energy loss, thereby reducing the EUE.

Contrastingly, the MD method utilized 20% more seeds while reducing total fertilizer application, which disrupted the nutrient balance essential for high-density rice planting. This imbalance resulted in early senescence of the functional leaves in rice and decreased the accumulation of photosynthetic assimilates during the grain-filling stage, reducing filled-grain percentage and grain weight, thereby lowering yield.^23^ Furthermore, the imbalance between fertilizer and seed inputs hindered the efficient utilization of fertilizers through agricultural mechanization, leading to excessive residual fertilizers in the soil, thereby increasing GHG emissions and energy losses.^24^ Consequently, although the MD method achieved higher yield and EBs through increased seeds while reduced fertilizer inputs, the imbalance led to escalating environmental burdens and decreased efficient use of energy, resulting in increased GHG emissions and reduced EUE.

In contrast to the MT method, the MAT method optimized the synergy among N, P, and K fertilizers by adjusting their application ratios, thereby ensuring balanced nutrition for rice plants, mitigating nutrient stress, and enhancing soil quality and microbial diversity.^25^^,^^26^ This improvement in soil health promoted nutrient cycling and facilitated more efficient absorption by rice roots, which, in turn, reduced soil fertilizer residues and their contribution to GHG emissions, thereby laying the foundation for higher yield formation. However, the lower seed rates associated with the MAT method resulted in reduced planting densities, limiting the effective use of nutrients by rice roots and causing nutrient surpluses that hindered further improvements in the production potential of the method. This led to fewer spikes per hectare, lower fruit set rates, and fewer grains per spike, thereby limiting the improvement of yield, EBs, and PFP, and ultimately resulting in lower comprehensive benefits for the MAT method. In contrast, the MAD method involved the highest total application of N, P, and K fertilizers at 543.13 kg/ha. This excessive fertilization disrupted the synergy among fertilizers and impeded root nutrient absorption, leading to reduced PFP. Over-application also left significant soil residues, exacerbating environmental pollution, increasing GHG emissions, compromising soil integrity, and raising rice susceptibility to pests and diseases.^27^^,^^28^ Consequently, the reduced yield led to low economic and energy outputs that failed to offset the high input costs, resulting in low EBs and EUE for the MAD method.

Therefore, the MT method sustained the highest productivity by utilizing increased application of N fertilizer and seed inputs, thereby improving yield, GHG emissions, and EBs to maximize comprehensive benefits. In contrast, the MD method’s reduced N fertilizer and increased seed inputs failed to meet the rice’s growth needs, leading to comparatively lower comprehensive benefits. The MAT method enhanced yield, EUE, and PFP while reducing GHG emissions through optimized application ratios of N, P, and K fertilizers, but the inherent limitations of this method reduced the ceiling on benefits, yielding lower comprehensive benefits. In contrast, the MAD method upsets balance between benefits due to the excessive use of N, P, and K fertilizers, culminating in the lowest comprehensive benefits.

### Pathways of achieving optimal benefits with the MT, MD, MAT, and MAD methods based on decision tree model

In Southwest China, MT, MD, MAT, and MAD methods typically demonstrate medium yield and low fertilizer productivity, making it crucial to identify and address the limitations impacting these key benefits. We categorized the conventional, suboptimal, and optimal benefits of various planting methods by dividing the benefit intervals at 1.2 and 0.8 times the mean values of each benefit (where the optimal or suboptimal benefits represent the maximum potential benefits achievable without adjusting agricultural inputs, i.e., “before optimization” with the models). A decision tree model was then used for path analysis to identify the key constraints preventing the transition from conventional to optimal or suboptimal benefit. Decision tree path analysis showed that an increase in yield, GHG emissions and EUE beyond 1.1 × 10^4^ kg/ha, 530.8 kg CO_2_-eq/ha, and 14 respectively in the MT method corresponded to a 21% probability of attaining the suboptimal benefits of HY-HG-HB-HP-LE. In contrast, in the MD method, increases in yield and EUE to 1.08 × 10^4^ kg/ha and 15 respectively, were linked to a 9% chance of reaching suboptimal benefits of HY-HG-HB-HP-LE. The MAT method demonstrated a 5% likelihood of achieving optimal benefits (HY-LG-HB-HP-HE) by elevating EUE, PFP, and EBs beyond 18, 18, and 1.9×10^4^ CNY/ha. However, the low yield limitations (yield ≤8.4 × 10^3^ kg/ha) of the MAD method make it difficult to achieve suboptimal or optimal benefits.

The fundamental reason limiting the improvement of benefits across distinct planting methods is the imbalance in agricultural input structures leading to conflicts among benefits. For instance, in the MT method, when N and K fertilizers applied at 165 kg/ha and 143 kg/ha respectively, and seed levels at 23 kg/ha, significantly enhanced yield by fostering N assimilation and photosynthesis of functional leaves, and subsequent assimilate accumulation of rice grains. This promoted spikelet differentiation and grain filling by supplying ample nutrients to meet the demands of high planting density and enriching soil nutrient to enhance its production capacity, which are crucial for yield augmentation.^29^ However, the utilization efficiencies for N and K fertilizers were only 34.8% and 50–60%, respectively, indicating that surplus N and K fertilizers inadvertently contribute to GHG emissions within the MT method (https://www.my478.com/). Given the complex relationship between yield, GHG emissions, and fertilizer application, surpassing suboptimal benefits remains a challenge in the MT method. In contrast, the suboptimal benefits of HY-HG-HB-HP-LE in the MD method were influenced by the interplay between yield and EUE arising from fertilizer inputs. The MD method only achieved suboptimal benefits (HY-HG-HB-HP-LE), primarily due to the interaction between the yield and EUE generated by N fertilizer and seed inputs. When N fertilizer and seed inputs were applied at 150 kg/ha and 33.7 kg/ha, respectively, enabling high yield and PFP, attributable to improved root morphology, thereby facilitating P and K interaction and absorption. However, the increased N input added to the system’s energy input burden and reduced the effective transfer of energy, thereby lowering the EUE.^30^ Furthermore, under high planting density, the relatively low levels of K fertilizer application weakened the nutrient absorption and utilization by the rice population, hindered the pathways of assimilate accumulation in grains and ultimately reduced yield.

In contrast, in the MAT method, optimal benefits (HY-LG-HB-HP-HE) are achieved when N fertilizer application is 143 kg/ha, and when P and K fertilizer applications are 83 kg/ha and 135 kg/ha, respectively. Reasonable supply of fertilizers enhanced soil nutrient cycling and efficient nutrient uptake by rice, thus optimizing yield.^31^^,^^32^ However, excessively low planting densities (seed amounts are 15 kg/ha) capped the method’s production potential. This indicates that optimal benefits do not always equate to the highest potential benefits. Meanwhile, the MAD method struggled to improve conventional benefits due to suboptimal yield. Excessive application of N, P, and K bound nutrients in the soil, risking soil salinization and decreasing nutrient availability.^33^^,^^34^ This, coupled with detrimental shifts in soil properties and microbial communities, constrained yield enhancement. Consequently, excessive use of N, P, and K fertilizers in the MAD method exacerbated the negative impacts, failing to improve conventional benefits.

In summary, in the MT method, the irrational pairing of N and K fertilizers with seed amounts results in only suboptimal benefits; in the MD method, the imbalance between N fertilizer and seed amounts also leads to suboptimal benefits; although the MAT method achieves optimal benefits, there remains significant potential for production if seed amounts are optimized; the MAD method maintains conventional benefits due to the imbalance in N, P, and K fertilizer application. Therefore, precisely optimizing the application ranges of agricultural inputs across different planting methods is key to maximizing production efficiency.

### Strategies for achieving better benefits with the MT, MD, MAT, and MAD methods based on random forest model and gradient boosting machine

Previous studies predominantly utilized one to three models to evaluate and predict datasets, often encountering issues such as limited robustness, poor generalization, and low predictive accuracy. In contrast, this study employs a hybrid approach, integrating multiple models and utilizing cross-validation between them, which significantly improves these problems, particularly model accuracy. Yet, this approach also increases the complexity of parameter tuning. We predicted and analyzed the effects of key agricultural inputs on the interaction of benefits across distinct planting methods through both models, identifying the agricultural input ranges under the optimal benefits (i.e., “after optimization” with the models). This provides the theoretical basis for precisely managing agricultural inputs in future field trials to maximize production efficiency. The specific optimization strategies are as follows: based on the suboptimal benefits of HY-HG-HB-HP-LE, the MT method could attain optimal benefits (HY-LG-HB-HP-HE) by reducing N fertilizer application to 139 kg/ha while increasing K fertilizer and seed inputs to 170 kg/ha, and 25 kg/ha, respectively. This strategic adjustment is projected to increase yield and EUE by 15% and 30% while decrease GHG emissions by 26%. Likewise, based on the suboptimal benefits of HY-HG-HB-HP-LE, the MD method could diminish GHG emissions by 21% and enhance EUE by 32% by reducing N fertilizer and seed inputs to 135 kg/ha and 30 kg/ha, boosting the achievement of optimal benefits. In the MAT method, increasing N and K fertilizers and seed inputs to 165 kg/ha, 175 kg/ha, and 20 kg/ha, could significantly elevate yield, EBs, PFP, and EUE by 7–14%, thus improving the probability to attain optimal benefits (HY-LG-HB-HP-HE). Additionally, in the MAD method, adjusting the N, P, and K fertilizer application ratio to 2:1:2 (with N fertilizer at 150 kg/ha), based on conventional benefits (MY-LG-LB-LP-LE), could increase yield and EBs by 33–40%, leading to the realization of suboptimal benefits (HY-HG-HB-LP-LE).

In the MT method, under the suboptimal benefits of HY-HG-HB-HP-LE, an increase in seed amounts and K fertilizer application, coupled with a reduction in N fertilizer application, aligns with the nutrient requirements of rice plants.^35^ This precise input optimization boosts an improved inter-root environment of rice, enhancing nutrient uptake and utilization by rice roots and consequently increasing fertilizer utilization efficiency. Meanwhile, this adjustment not only satisfies the high nutrient demands of rice populations at elevated planting densities but also promotes robust plant growth and enhanced assimilate accumulation in rice grains, facilitating an increase in panicle numbers, grain filling, and grains per spike, thereby improving yield.^36^^,^^37^ Additionally, optimizing K fertilizer application enhances the photosynthetic efficiency of functional leaves, promoting efficient assimilate distribution to rice grains, crucial for achieving high yield.^31^^,^^38^ This precise adjustment also supports a beneficial increase in the energy output-to-input cost ratio, thereby improving EUE. Importantly, this precise matching of agricultural inputs aligns with expected environmental objectives by minimizing fertilizer residues and GHG emissions without compromising yield.^39^ Likewise, in the MD method, precise adjustments, such as appropriate reducing N fertilizer application and seed amounts can relatively alleviate nutrient shortages, optimize the carrying capacity of the production system, and support effective assimilate accumulation during the grain filling process, conducive to high yield.^40^^,^^41^ Similar to MT method, reducing N fertilizer application in the MD method decreases the primary source of GHG emissions, enhancing EUE and still maintaining high yield levels without significant yield loss.^42^ The MAT method increases rice population density by augmenting seed amounts by 33%, which enhances canopy photosynthesis and promotes assimilate synthesis, and by adjusting fertilizer application—increasing N and K fertilizer application, thereby improving soil nutrient availability, nutrient utilization efficiency, and supporting increases in effective tiller numbers and grain counts per spike, thereby laying the foundation for higher yield.^43^ The MAD method employs a balanced N, P, and K fertilizer ratio of 2:1:2 to improve soil conditions and nutrient cycling, fostering higher rice production capacities,^44^ thereby attaining suboptimal benefits of HY-HG-HB-LP-LE by improving yield and EBs.

Therefore, utilizing decision-making systems supported by machine learning can enhance the precision of agricultural input management across different planting methods, improve environmental benefits by maximizing rice production efficiency, and thus maintain precise, efficient and sustainable rice production in Southwest China. Yet, applying these methods to other regions worldwide requires careful consideration of model and data uncertainties. The primary sources of uncertainty include regional differences in climate, soil fertility, and agricultural practices (particularly the extent of agricultural mechanization). These factors often limit rice yield improvement and hinder the effective implementation of agricultural practices. In order to reduce the uncertainty caused by these factors, it is essential to further enhance model accuracy through extensive data training. Notably, the accuracy of these models largely depends on the quality of the training data. However, the availability and quality of data across different regions vary, especially in some developing countries, where the precision and coverage of data collection are often limited. Therefore, the development of a global agricultural input-output database could address the issue of inconsistent data availability and quality across regions, thereby improving the predictive accuracy and applicability of the models. In conclusion, model training, combined with the development of a global database, can more effectively reduce model and data-related uncertainties.

### Conclusion

The MT method demonstrated superior comprehensive benefits, surpassing those of the MD, MAT, and MAD methods by factors of 1.1, 2.1, and 2.6, respectively. Based on the suboptimal benefits, increasing the K fertilizer application and seed amounts while reducing the N fertilizer application enhanced the yield and EUE while decreased GHG emissions in the MT method; similarly, reducing the N fertilizer application and seed amounts in the MD method also enhanced the EUE and decreased GHG emissions. Together, these changes achieved optimal benefits (HY-LG-HB-HP-HE) for both methods. In the MAT method, further increases in N and K fertilizers and seed amounts, based on optimal benefits, resulted in improvements in yield, EBs, PFP, and EUE, thereby attaining higher comprehensive benefits. Additionally, in the MAD method, adjusting the N, P, and K fertilizer ratio to 2:1:2 under conventional benefits improved yield and EBs, achieving suboptimal benefits (HY-HG-HB-LP-LE). Additionally, although the MT method exhibited higher GHG emissions and lower EUE, its production potential remained the highest. By further optimizing the relations between N and P fertilizers and seed amounts through the decision-making system, this method’s comprehensive benefits could be significantly enhanced, making it the most valuable for widespread application in agricultural production. This study provides insights into how strategic adjustments to agricultural inputs can maximize agricultural efficiency and sustainability across four rice planting methods.

### Limitations of the study

This study focused on a single rice production system and did not systematically consider the production systems of single-cropping, double-cropping, or ratoon rice. Moreover, the models were exclusively driven by data derived from the actual rice growing process, without considering meteorological data. This omission limits the potential for time series analysis and may restrict the generalizability of the findings across varied climatic conditions.

## Resource availability

### Lead contact

Further information and requests for resources should be directed to and will be fulfilled by the lead contact Zongkui Chen (chenzongkui90@foxmail.com).

### Materials availability

This study did not generate new unique reagents.

### Data and code availability

• The mapping data and computer code in the manuscript have been uploaded at Zenodo.
• This paper does not report original code.
• Any additional information required to reanalyze the data reported in this paper is available from the lead contact upon request.

## Acknowledgments

This work was supported by 10.13039/501100002858China Postdoctoral Science Foundation (2022M722301), Sichuan Province Innovative Talent Funding Project for Postdoctoral Fellows (BX202207) and 10.13039/501100018542Sichuan Natural Science Foundation of China (2023NSFC0014).

## Author contributions

Conceptualization, Z.C., J.M., Xiafei L., Y.S., and Z.Y.; data curation, T.L., Xiafei L., Xinrui L., Z.W., H.Y., Y.M., R.L., Y.L., Z.L., P.D., and Z.P.; roles/writing - original draft, T.L.; writing - review and editing, Z.C. and Xiafei L.

## Declaration of interests

The authors declare no competing interests.

## STAR★Methods

### Key resources table

**Table:** 

REAGENT or RESOURCE	SOURCE	IDENTIFIER
**Deposited data**		

Mapping data and computer code	Zenodo	https://zenodo.org/records/14211248

**Software and algorithms**		

GetData graphic digitizing	GetData graphic digitizing software	http://www.getdata-graph-digitizer.com
Simapro 9.0	Simapro software	https://simapro.com/
R software version 4.3.1	R Core Team	https://www.r-project.org/
Origin v.2021	OriginLab	https://www.originlab.com/
SPSS v.26.0	IBM SPSS software	https://www.ibm.com/spss

### Experimental model and study participant details

This study does not include experiments or subjects.

### Method details

#### Study site

The study site is located at 97°E−110°E, 21°N-34°N, and mainly includes Sichuan, Yunnan, and Guizhou Provinces, and Chongqing City, Southwest China (Figure S1; Table S1). It is characterized by basins and hills. Southwest China has a subtropical monsoon climate, with annual average temperatures of 13.12°C–14.09°C and annual precipitation of 954.33–2306.93 mm. The rice growing season typically extends from the middle ten days of May to the middle ten days of October. The soil contents of organic matter, total N, total P, and total K are 21.6–43.5, 1.6–2.6, 1.25–2.18 and 9.87–15.48 g kg^−1^, respectively. The soil contents of available N, available P and available K are 103.19–181.34, 22.4–43.76 and 79.5–170.48 mg kg^−1^, respectively. The soil pH is 6.2–6.7.^43^

#### Data sources

This study, based on field experiments conducted in the Southwest region, selected agricultural inputs (e.g., fertilizer application and seed amounts) under different planting methods to construct a rice production decision model. This model optimizes agricultural inputs to achieve higher input conversion efficiency and yield, while also reducing GHG emissions in rice systems. Data were collected from peer-reviewed publications issued between January 2010 and June 2022 on MT, MD, MAT, and MAD methods used in Southwest China, including Sichuan, Yunnan, and Guizhou Province, and Chongqing City. These publications were searched using terms such as "mechanical transplanting or direct seeding", "manual transplanting or direct seeding", "yield", and "rice", through the Web of Science (http://apps.webofknowledge.com/) and China National Knowledge Infrastructure (http://www.cnki.net/) databases. The search for peer-reviewed publications should meet the following standards: (1) the field experiments were carried out at sites in Southwest China (excluding pot and greenhouse gas experiments); (2) the input information such as the amounts of N, P and K fertilizers and seed, pesticides (herbicides and insecticides) were included, and the studies also investigated and measured the duration of manual or mechanical involvement in agricultural activities using the different planting methods to subsequently calculate the economic and energy input and output; and (3) the processing conditions were not were unrestricted, and the same data were not duplicated in multiple articles, and at least one year of trial data were included. In addition, we used GetData graphic digitizing software (http://www.getdata-graph-digitizer.com/) to extract the digitized image data. From this rigorous process, 302, 202, 139, and 1079 datasets were obtained for MT, MD, MAT, and MAD methods, respectively. Finally, based on comprehensive benefits assessment and analysis of agricultural input optimization using machine learning, we also created a logic diagram to clearly and briefly show the entire research process (see below figure), and life cycle assessment of agricultural inputs and output inputs of distinct planting methods (Figure S2). Furthermore, all abbreviations used in this study can be found in Table 1.

**Figure:**
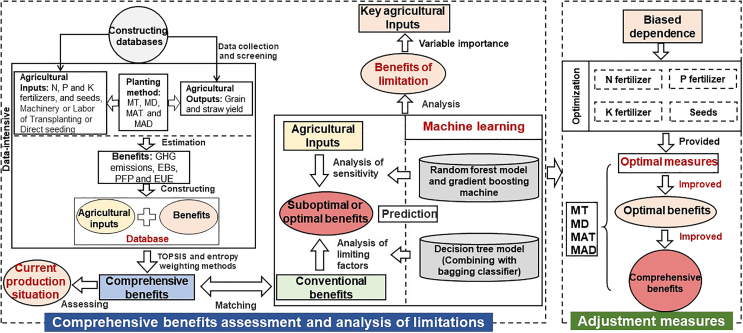
Logic diagram Comprehensive benefits assessment and analysis of agricultural input optimization using machine learning (including data collection and database construction, comprehensive benefits assessment, analysis and machine learning integration and optimization and adjustment measures).

**Table 1:** List of abbreviations

Abbreviations	Definition
MT	Mechanical transplanting
MD	Mechanical direct seeding
MAT	Manual transplanting
MAD	Manual direct seeding
GHG	Greenhouse gas
EBs	Economic benefits
PFP	Partial fertilizer productivity
EUE	Energy use efficiency
TOPSIS	Technique for Order Preference by Similarity to an Ideal Solution
LY	Low yield
MY	Medium yield
HY	High yield
LG	Low greenhouse gas
HG	High greenhouse gas
LB	Low economic benefits
HB	High economic benefits
LP	Low partial fertilizer productivity
HP	High partial fertilizer productivity
LE	Low energy use efficiency
HE	High energy use efficiency

#### Estimation of EBs

The EBs were expressed as the difference between total returns minus total inputs. The agricultural inputs were calculated from the economic coefficients of all agricultural inputs, including N, P, and K fertilizers, seeds, herbicides, pesticides, labor and machinery, and the total return was calculated based on the prevailing market price of grain yield. The unit sale prices of the agricultural inputs and grain are shown in Table 2, and the following equation was then used to determine the economic output and EBs^45^:

(Equation 1) Economicoutput=Grainyield×Salepriceperunit

(Equation 2) EBs=Economicoutput-Inputcosts

**Table 2:** Carbon emission coefficients, energy equivalents, and costs of various agricultural inputs in rice

Items	Units	Cost (CNY/ha)	GHG emissions coefficients (kg CO_2_-eq/ha)	Energy equivalents （MJ/ha）
**Agricultural inputs**				

Nitrogen (N) fertilizer	kg	3.8	1.3	60.6
Phosphate (P_2_O_5_) fertilizer	kg	1.25	0.2	11.93
Potassium (K_2_O) fertilizer	kg	2	0.2	6.7
Herbicides	kg	175	6.30	238
Pesticides	kg	100	5.10	199
Rice seed	kg	55	–	17
Labor-MAT	h	150	0	214.95
Labor-MAD	h	150	0	127.5
Mechanization-MT	h	120	0.07	37.5
Mechanization-MD	h	120	0.07	34.95

**Agricultural outputs**				

Rice grain	kg	2.7	–	14.7
Rice straw	kg	–	–	12.5

CNY, Chinese Yuan.

#### Calculation of GHG emissions

The GHG emissions from rice production mainly originate from the emissions caused by agricultural inputs such as fertilizers, pesticides and agricultural machinery. The emission coefficients of various agricultural inputs considered for the rice planting methods are shown in Table 2 and were generally determined by summing the emissions from each agricultural input and multiplying them by their emission coefficient, using the following calculation formula (where ${“R}_{i}$” is the amount of different agricultural inputs and $“D_{i}’’$ is the corresponding emission coefficient)^46^:

(Equation 3) $$\text{GHG}\;\text{emissions}=\sum \left(R_{i}\times D_{i}\right)$$

#### Calculation of PFP

The PFP in this study was calculated based on the ratio of grain yield to that of total fertilizer input according to the following formula^47^:

(Equation 4) $$\text{PFP}=\frac{\text{Grain}\;\text{yield}}{\text{Total}\;\text{fertilizer}\;\text{inputs}}$$

#### Calculation of EUE

Sources of energy input included N, P, K, seeds, herbicides, insecticides, labor and machinery, and sources of energy output included grain yield and straw yield. To calculate the EUE of this study, the input and output of agriculture were converted into energy input and output by multiplying them by their respective energy equivalents (Table 2).^48^

(Equation 5) $$\text{EUE}=\frac{\text{Total}\;\text{Energy}\;\text{Output}\;\left(\text{MJ}/\text{ha}\right)}{\text{Total}\;\text{Energy}\;\text{Input}\left(\text{MJ}/\text{ha}\right)}$$

#### Ranges for high, medium and low yield and high or low GHG emissions, EBs, PFP and EUE

The definition of super high yield rice was proposed based on a 20% increase in the average rice grain yield,^49^ and we defined the high, medium and low yield and high or low GHG emissions, EBs, PFP and EUE in each planting methods. As follows: High yield: ≥1.2 × average yield; range of medium yield: 0.8 × average yield to 1.2 × average yield; low yield: ≤0.8 × average yield. High GHG emissions: ≥1.2 × average GHG emissions; low GHG emissions: <1.2 × average GHG emissions. High EBs ≥1.2 × average EBs; low EBs: <1.2 × average EBs. High PFP ≥1.2 × average PFP; low PFP: <1.2 × average PFP. High EUE ≥1.2 × average EUE; low EUE: <1.2 × average EUE (Table 3).

**Table 3:** Yield, greenhouse gas (GHG) emissions, partial fertilizer productivity (PFP), energy use efficiency (EUE), and economic benefits (EBs) in mechanical transplanting (MT) (*n* = 302), mechanical direct seeding (MD) (*n* = 202), manual transplanting (MAT) (*n* = 1,079) and manual direct seeding (MAD) (*n* = 139) methods

Items	MD	MT	MAD	MAT
The range of middle yield (kg/ha)	7227.1–10840.7			
Low limit of high GHG emissions (kg CO_2_-eq/ha)	386.04			
Low limit of high EBs (CNY/ha)	18567.4			
Low limit of high PFP	29.29			
Low limit of high EUE	18.31			
Yield (kg/ha)				
Average	9268.6	10033.2	8135.9	8839.7
High	11346.4 a	11607.9 a	10862.8 b	11708.5 a
Medium	9311.1 a	9457.4 a	8442.1 c	8996.9 b
Low	6865.6 a	6154.4 a	5692.5 a	6054.4 a
GHG emissions (kg CO_2_-eq/ha)				
Average	490.00	514.61	420.45	326.15
High	523.17 b	535.03 a	518.32 b	536.23 a
Low	337.00 b	384.62 a	336.94 b	320.76 b
EBs (yuan/ha)				
Average	18834.1	20043.3	12464.9	13986.7
High	20706.8 ab	21937.2 a	19416.1 b	20967.8 a
Low	15729.2 a	16159.9 a	12259.0 b	12824.4 b
PFP				
Average	26.94	28.51	16.39	24.00
High	33.37 a	32.38 a	–	34.39 a
Low	25.20 a	26.04 a	16.39 c	21.96 b
EUE				
Average	13.91	14.49	11.27	15.90
High	20.17 a	26.12 a	22.04 a	25.11 a
Low	12.82 b	13.49 a	10.95 c	13.59 b

If both lowercase letters are the same, then the difference between the various treatments is not significant (*p* > 0.05); otherwise, it is significant (*p* < 0.05).

#### Structural equation model

Structural equation models for path construction and non-standardized correlation analysis were performed using an analysis of Moment Structures. There are five dependent variables in the theoretical framework of the study: Rice yield, GHG emissions, EBs, PFP and EUE. The path analysis between each agricultural input and five benefits was established through structural equation model, and the path coefficients “r” (0 ≤ r ≤ 1) were used to describe the relationship between each variable and the dependent variable for subsequent analysis.^50^

#### Life cycle analysis

Based on path analysis of structural equation model, we inputted the agricultural inputs and outputs of different planting methods into the Simapro software (https://simapro.com/) for Life Cycle analysis to generate KPI values, which were then used to evaluate and compare different benefit levels (conventional benefits versus suboptimal or optimal benefits).

#### Decision tree model

We constructed a decision tree model based on the ID3 algorithm to perform path analysis (from conventional to optimal benefits) of benefit combinations across distinct planting methods and predict the probability of achieving optimal benefits. In this process, we matched the conventional benefits set in the model with the actual comprehensive benefits of distinct planting methods for prediction.^51^ Initially, 70% of the data is randomly selected as the training set, and 30% as the test set. An ID3 decision tree is constructed using the training set, and pruning is applied to prevent overfitting or excessive complexity of the model. Subsequently, the rpart.plot function is employed to create various visualizations of the trees, aiding in the explanation of the model structure and decision paths. Finally, the model is evaluated using training set data, and a confusion matrix (comprising the sum of True Positives and False Positives, i.e., Detection Prevalence) and accuracy are calculated to assess model performance.

#### Bagging classifier

To enhance the predictive reliability of the decision tree model through cross-validation, this study integrates a bagging classifier to enhance the accuracy of the decision tree model (Tables S1 and S2). We utilized the packages of “ipred” for bagging implementation and the rpart.plot function for decision tree visualization.^52^ The dataset was split into 75% training data and 25% testing data using stratified sampling. The training set was used to build a Bagging model, and a confusion matrix was used to evaluate the model’s predictions on the test data to assess accuracy and other performance metrics.

#### Random forest model

The random forest model developed by Breiman is a machine learning method that combines classification trees, regression trees, and repeated sampling with putbacks.^53^ Based on optimal benefits achieved only at present, the random forest model was used to construct decision trees to predict the optimization strategies for agricultural inputs of achieving different rice plant methods in this study. This study used a self-service repeated sampling method to split the dataset into a 3:1 ratio for training and testing sets. The training set data were selected, and the randomForest function was used to build, predict and train the model while calculating the importance of each agricultural input on benefits. The testing set was used to evaluate the predictive performance of each final trained model. During this process, we selected the optimal number of trees (ntree) and the number of variables at each node (mtry) through multiple simulations and cross-validation to reduce overfitting and enhance the model’s predictive accuracy, generalization ability, and interpretability of key variables. Additionally, we employed gradient boosting machine for cross-validation to further enhance the accuracy of the predictions.

#### Gradient boosting machine

This study also employs gradient boosting machine through decision trees to forecast optimized strategies for agricultural inputs in various rice planting methods. The training dataset is selected and the model constructed using the gbm function, with the optimal iteration determined by the gbm.perf function based on cross-validation. The testing set was used to evaluate the performance of each final trained model.^54^

#### Mixed effects model

Based on the above two models, this study also incorporates mixed-effects model to further reveal the response of the dependent variable to predictive factors, thereby further improving the accuracy of predicting the importance of key agricultural inputs (Figure S3). Specifically, we employed a mixed-effects model to analyze the effects of agricultural inputs on yield,^55^ GHG emissions, EBs, PFP and EUE. We imported the dataset and standardized continuous variables, except for the response variable "benefits" and the random effect "Block," to ensure comparability across predictors. Using the lmer function from the lmerTest package, we fitted a mixed-effects model with "N," "P_2_O_5_," "K_2_O," and "Seed" as fixed effects and "Block" as a random effect (i.e., four planting methods). A forest plot was created to visualize the estimates and confidence intervals of the model’s predictors, highlighting the effects of each input on benefits.

#### Model evaluation

To assess the accuracy of the models, the decision coefficient (R^2^), root-mean-square error (RMSE), mean absolute error (MAE) and relative root-mean-square error (rRMSE) were used as evaluation indexes.^56^ The equations for the three accuracy metrics are as follows:

(Equation 6) $$R^{2}=1-\frac{\sum_{i=1}^{n}{\left(y_{i}-y_{i}^{\prime }\right)}^{2}}{\sum_{i=1}^{n}{\left(y_{i}-\bar{y}\right)}^{2}}$$

(Equation 7) $$\text{RMSE}=\sqrt{\frac{\sum_{i=1}^{n}{\left(y_{i}-y_{i}^{\prime }\right)}^{2}}{n}}$$

(Equation 8) $$\text{rRMSE}=\frac{\text{RMSE}}{\bar{y}}\times 100\%$$

### Quantification and statistical analysis

This study primarily employed a random forest model and gradient boosting machine to optimize agricultural benefits by their varying agricultural input in the four rice planting methods, where n represents the sample size for each planting method in the Southwest region, specifically, the *n* values for MT, MD, MAT, and MAD methods were 302, 202, 1079, and 139, respectively (Figures 1, 2, 3, 4, 5, 6, 7, 8, and 9; Table 3). The mean, median, and standard deviation values are shown in Figure 3.

The plots “ggplot2” in R version 4.0.2 (https://cran.r-project.org/) was utilized for graphing and the packages (“skimr”, “caret”, “rpart”, “randomForest” and “gbm”) were used to implement decision tree and random forest models and gradient boosting machine. Analysis of variance (ANOVA) followed by Tukey’s honestly significant difference (HSD) test was performed using SPSS v.26.0 (IBM Corporation, Armonk, NY, USA). Differences between rice planting methods were considered significant at *p* < 0.01 and *p* < 0.05. Gray relational analysis (GRA) was also performed using SPSS v.26.0. Figures were plotted using Origin v.2021 (Origin Lab Corp, Northampton, MA, USA). Life cycle analysis was conducted using Simapro 9.0 (PRé Sustainability B.V., Amersfoort, Netherlands).
