# Supplementary material for: Utilizing machine learning to optimize agricultural inputs for improved rice production benefits
Source: iScience. 2024 Nov 16;27(12):111407. doi: 10.1016/j.isci.2024.111407 (PMC11648248; doi:10.1016/j.isci.2024.111407)
Supplement: Document S1. Figures S1–S3 and Table S1–S3 [file mmc1.pdf]

iScience, Volume *27*

Supplemental information

Utilizing machine learning to optimize agricultural inputs for improved rice production benefits

Tao Liu, Xiafei Li, Xinrui Li, Zhonglin Wang, Huilai Yin, Yangming Ma, Yongheng Luo, Ruhongji Liu, Zhixin Li, Pengxin Deng, Zhenglan Peng, Zhiyuan Yang, Yongjian Sun, Jun Ma, and Zongkui Chen

Study area

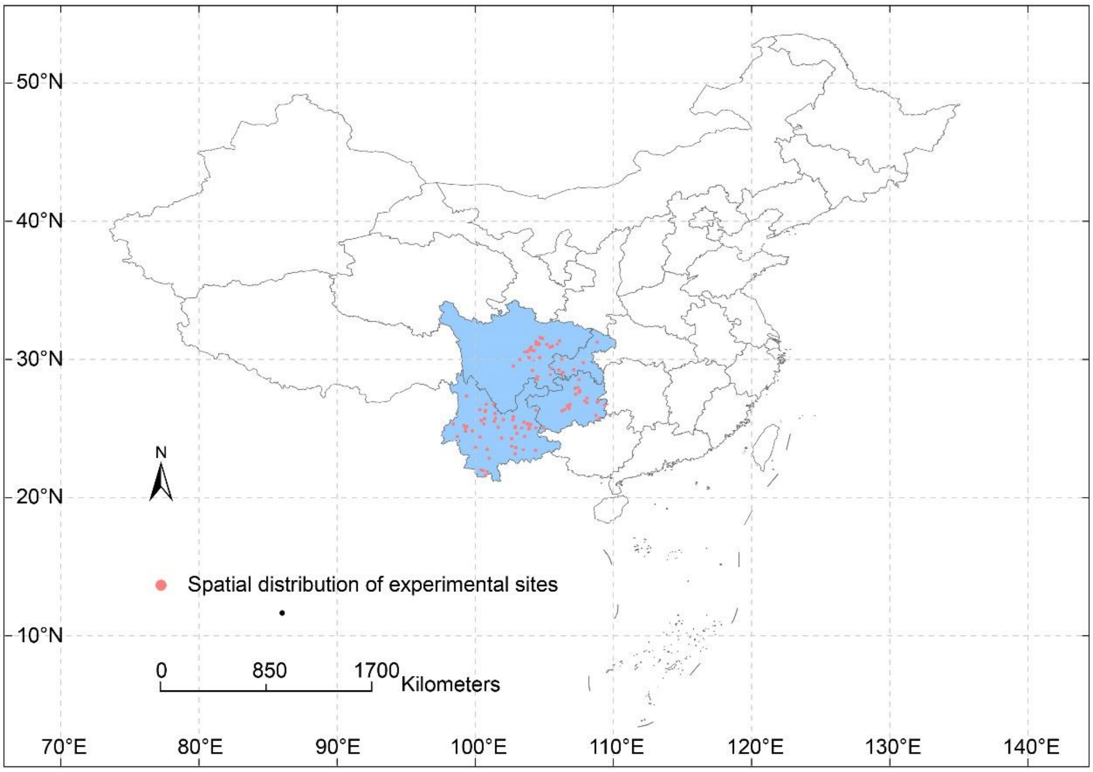

Figure S1. Map of the study area.

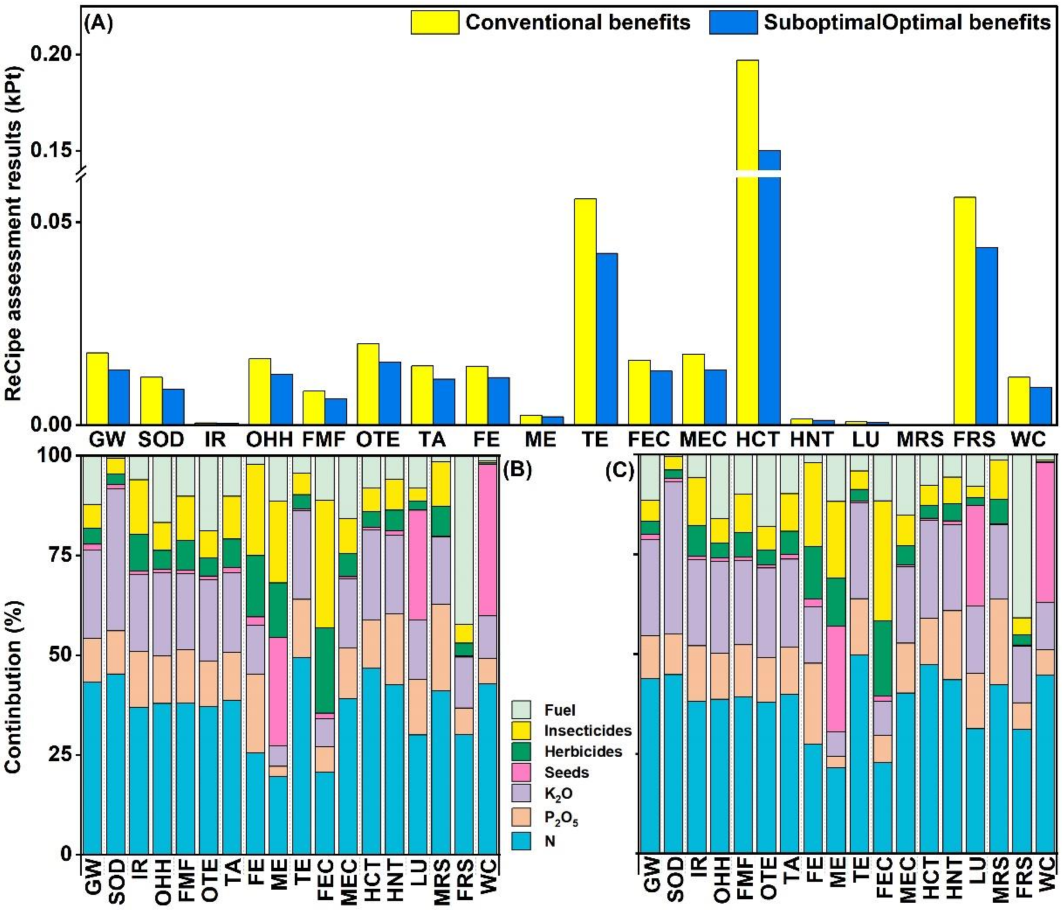

Figure S2. The Life Cycle Assessment of distinct benefit levels, related to Figure 4. Note: A compares the analysis of various KPI values under conventional and suboptimal or optimal benefits. B and C respectively illustrate the contributions of different KPI values (including Global warming (GW), Stratospheric ozone depletion (SOD), Ionizing radiation (IR), Human health (OHH), Fine particulate matter formation (FMF), Terrestrial ecosystems (OTE), Terrestrial acidification (TA), Freshwater eutrophication (FE), Marine eutrophication (ME), Terrestrial ecotoxicity (TE), Freshwater ecotoxicity (FEC), Marine ecotoxicity (MEC), Human carcinogenic toxicity (HCT), Human non-carcinogenic toxicity (HNT), Land use (LU), Mineral resource scarcity (MRS), Fossil resource scarcity (FRS) and Water consumption (WC)) to conventional and suboptimal or optimal benefits.

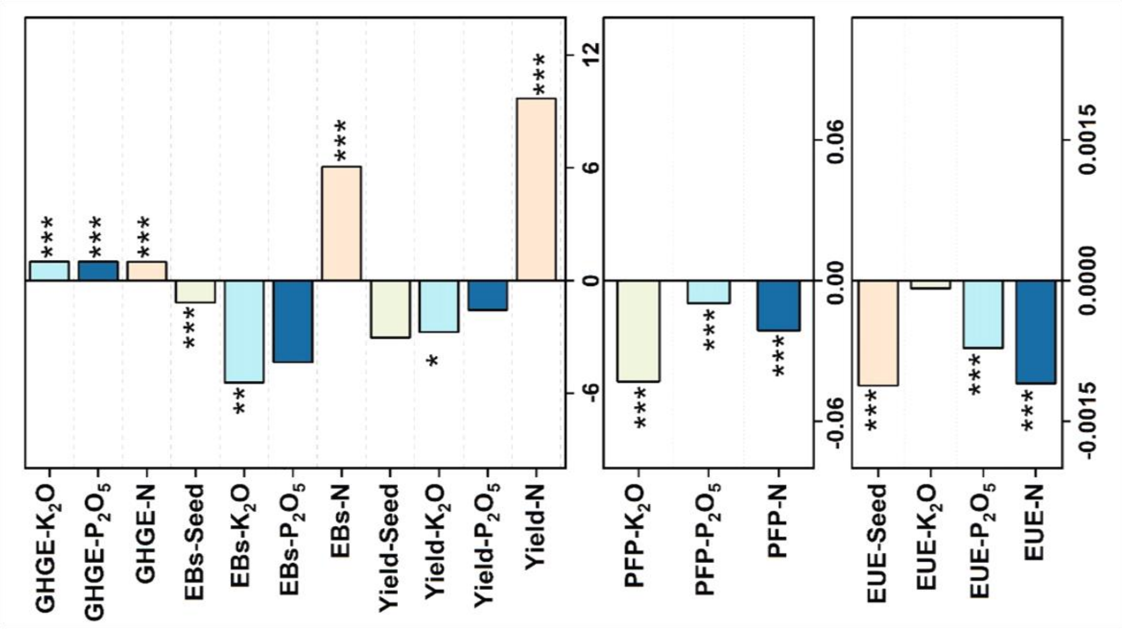

Figure S3. The extent of the effect of benefits on agricultural inputs, related to Figure 8. Note: The extent of the effect of Yield, economic benefits (EBs), greenhouse gas emissions (GHGE), partial fertilizer productivity (PFP) and energy use efficiency (EUE) on agricultural inputs in mechanical transplanting (MT) (n=302), mechanical direct seeding (MD) (n=202), manual transplanting (MAT) (n=139) and manual direct seeding (MAD) (n=1079) methods.

Table S1. Information on rice production in the Southwest China.

Area	Application of agricultural machinery (10^4 kW)	Rice planting area (10^3 ha)	Rice yield (10^4 t)
Sichuan	4923.33	1874.00	1462.28
Chongqing	1565.60	659.19	485.24
Yunnan	2913.65	709.46	464.67
Guizhou	2805.71	613.77	395.00
National total	110597.19	29450.11	20849.48

Table S2. Detection Prevalence (Sum of Ture Positives and False Positives) in the bagging classifier, related to Figure 5.

Benefits combination	MT	MD	MAT	MAD
LY-LG-LB-LP-LE	-	-	0.09	-
LY-HG-LB-LP-LE	-	-	0.01	0.16
LY-LG-LB-LP-HE	-	0.05	0.05	-
MY-LG-LB-LP-LE	0.04	-	0.59	-
MY-LG-LB-LP-HE	-	-	0.03	-
MY-LG-LB-HP-HE	-	0.11	0.07	-
MY-LG-HB-LP-LE	-	-	0.03	
MY-HG-LB-LP-LE	0.20	0.22	0.02	0.84
MY-LG-LB-HP-HE	0.07	-	0.07	-
MY-HG-HB-LP-LE	0.31	0.54	-	-
MY-HG-HB-HP-LE	0.07	-	-	-
HY-LG-HB-LP-LE	-	-	0.05	-
HY-LG-HB-HP-HE	-	-	0.05	-
HY-LG-HB-HP-LE	-	-	0.02	-
HY-HG-HB-LP-LE	0.09	-	-	-
HY-HG-HB-HP-LE	0.21	0.09	-	-

Table S3. Accuracy of bagging classifier and decision tree model, related to Figure 5.

Treatment	Bagging classifier	Decision tree model
MT	0.971	0.901
MD	0.891	0.915
MAT	0.992	0.996
MAD	0.945	0.958
